# Phosphodiesterases Expression during Murine Cardiac Development

**DOI:** 10.3390/ijms22052593

**Published:** 2021-03-05

**Authors:** Thays Maria da Conceição Silva Carvalho, Silvia Cardarelli, Mauro Giorgi, Andrea Lenzi, Andrea M. Isidori, Fabio Naro

**Affiliations:** 1 Department of Anatomical, Histological, Forensic and Orthopedic Sciences, Sapienza University, 00161 Rome, Italy; thaysmariadaconceiao.silvacarvalho@uniroma1.it (T.M.d.C.S.C.); silvia.cardarelli@uniroma1.it (S.C.); 2 Department of Biology and Biotechnology “C. Darwin”, Sapienza University, 00185 Rome, Italy; mauro.giorgi@uniroma1.it; 3 Department of Experimental Medicine, Sapienza University, 00161 Rome, Italy; andrea.lenzi@uniroma1.it (A.L.); andrea.isidori@uniroma1.it (A.M.I.)

**Keywords:** cyclic nucleotides, heart development, phosphodiesterases

## Abstract

3′-5′ cyclic nucleotide phosphodiesterases (PDEs) are a large family of enzymes playing a fundamental role in the control of intracellular levels of cAMP and cGMP. Emerging evidence suggested an important role of phosphodiesterases in heart formation, but little is known about the expression of phosphodiesterases during cardiac development. In the present study, the pattern of expression and enzymatic activity of phosphodiesterases was investigated at different stages of heart formation. C57BL/6 mice were mated and embryos were collected from 14.5 to 18.5 days of development. Data obtained by qRT-PCR and Western blot analysis showed that seven different isoforms are expressed during heart development, and PDE1C, PDE2A, PDE4D, PDE5A and PDE8A are modulated from E14.5 to E18.5. In heart homogenates, the total cAMP and cGMP hydrolytic activity is constant at the evaluated times, and PDE4 accounts for the majority of the cAMP hydrolyzing ability and PDE2A accounts for cGMP hydrolysis. This study showed that a subset of PDEs is expressed in developing mice heart and some of them are modulated to maintain constant nucleotide phosphodiesterase activity in embryonic and fetal heart.

## 1. Introduction

Heart development is a complex process which requires an interplay of different transcription factors, signaling pathways and morphological modifications [1]. Mouse heart formation begins at embryonic day 7.5 (E7.5) when a group of mesodermal cells migrates to the midline, forming the cardiac crescent [1]. At E8.5, the cardiac crescent changes its conformation, forming the linear heart tube, cardiac looping occurs at E10.5, atrioventricular septation and trabeculation occurs at E12.5 and a four-chambered heart is formed at E14.5 [1]. At E15.5 and E16.5, the atrioventricular valve leaflets and the coronary arteries are modified, achieving the definitive conformation, and at E18.5, the definitive external prenatal heart configuration is completed [2].

The formation of the heart is tightly controlled by a set of signal transduction pathways including the intracellular second messenger cyclic adenosine monophosphate (cAMP) and cyclic guanosine monophosphate (cGMP) [3]. The intracellular levels of cyclic nucleotides are regulated by the balance between their synthesis and degradation. cAMP is synthesized by adenylyl cyclases through agonist binding to G-protein-coupled receptors. cGMP is produced by a soluble guanylyl cyclase activated by nitric oxide and a particulate guanylyl cyclase activated by the natriuretic peptides [4]. Both cyclic nucleotides are hydrolyzed by the 3′5′cyclic nucleotides phosphodiesterases (PDEs) into their inactive forms 5′-AMP and 5′-GMP.

Eleven PDEs have been described and they are defined by their cellular functions, catalytic properties, protein structure and affinity for cAMP and cGMP [5]. Seven PDEs are expressed in the adult heart: PDE1, PDE2 and PDE3 which hydrolyze both cAMP and cGMP; PDE4 and PDE8 which hydrolyze cAMP; and PDE5 and PDE9 which are cGMP specific [6]. Despite the important role of the cAMP–cGMP/PDEs pathway in cardiac processes like contraction/relaxation, cell differentiation, proliferation and survival [7], very few data are available regarding PDEs’ function in cardiac development. Recently, it was reported that there are seven PDE mRNAs expressed in the murine heart at different stages of development [8] and, more importantly, it was demonstrated that PDE2A plays a critical role in heart development because its genetic ablation induces congenital defects [9]. Table 1 reports current data, obtained in transgenic animal model, on PDE isoforms involved in cardiac functions.

Considering these observations, the present study was carried out to further characterize the pattern of PDE expression in cardiac development at mRNA, protein and activity levels. This information will provide a useful tool to clarify the role of PDEs in the framework of the cardiac development and their potential role in cardiac diseases.

## 2. Results

### 2.1. PDE mRNA Expression Levels Vary between Embryonic and Fetal Cardiac Development

**Figure 1:**
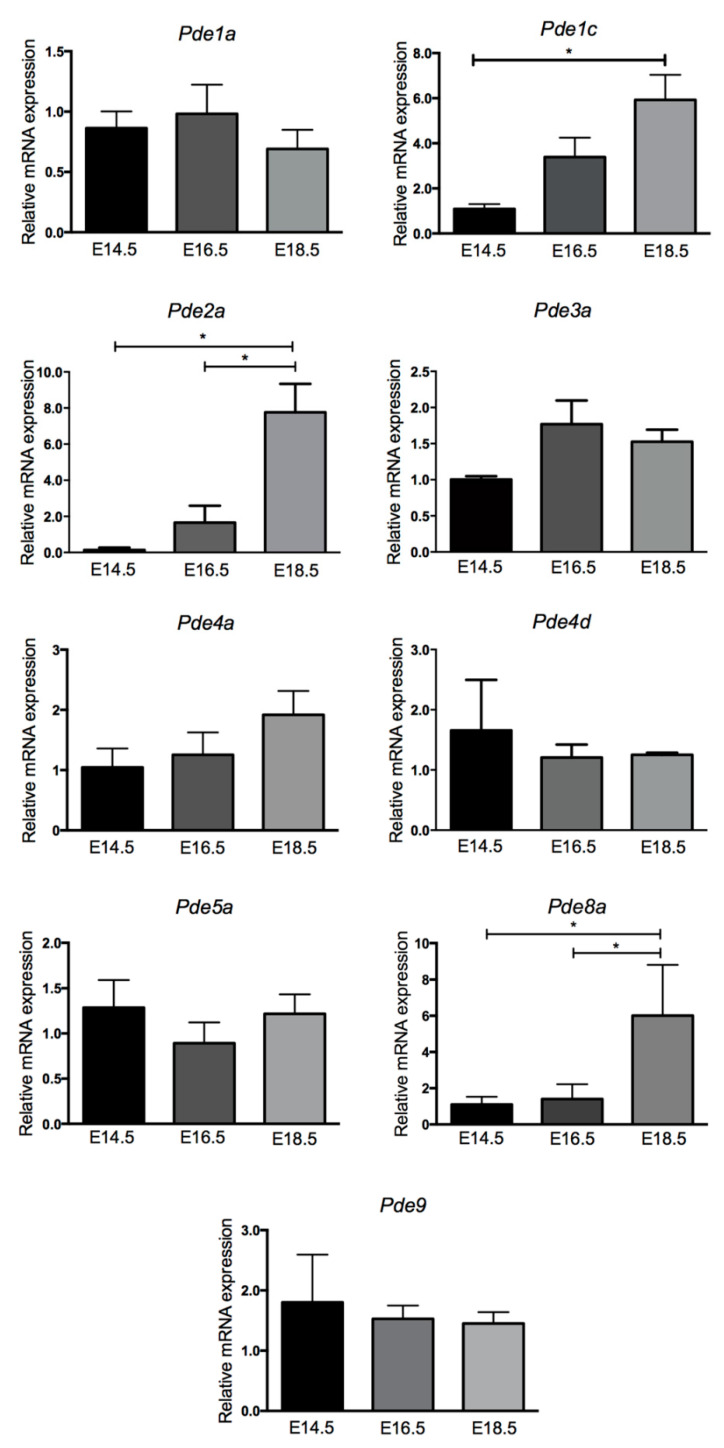
PDE gene expression during heart formation. qRT-PCR analysis of *Pde1a*, *Pde1c*, *Pde2a*, *Pde3a*, *Pde4a*, *Pde4d*, *Pde5a*, *Pde8a* and *Pde9* mRNA expression in E14.5, E16.5 and E18.5 mouse hearts. The relative expression was normalized versus *Gapdh* gene. Data represent the mean ± SD of five hearts for each stage. One-way ANOVA was used for statistical analysis. * *p* < 0.05.

It was previously shown by semiquantitative reverse transcriptase polymerase chain reaction (RT-PCR) that the mRNAs of *Pde1*, *Pde2*, *Pde3*, *Pde4*, *Pde5*, *Pde8* and *Pde9* were present in the heart of embryonic and fetal mice [8]. In order to detect PDE expression changes in the developing heart, the mRNA levels of selected variants of these PDEs were measured by qRT-PCR at E14.5, E16.5 and E18.5 (Figure 1).

The *Pde1* gene encodes for three (*1a*, *1b* and *1c*) Ca^2+^/calmodulin (CaM)-stimulated phosphodiesterase activities, all able to hydrolyze both cAMP and cGMP [18]. Quantitative expression analysis showed that mRNA of *Pde1a* is present at similar levels at all stages of heart development. In contrast, *Pde1c* mRNA was expressed at very low levels at E14.5 and greatly increased expression at E16.5, reaching statistical significance at E18.5.

PDE2A is a cAMP and cGMP hydrolyzing activity stimulated by cGMP. *Pde2a* transcript, required for normal heart development [9], is expressed at low levels at E14.5 and showed a gradual increase over time with expression being significantly greater at E18.5.

PDE3A, comprising the 3A and 3B isoforms [12], is a member of the cGMP-inhibited phosphodiesterase, hydrolyzing both cAMP and cGMP.

*Pde3a* mRNA was moderately expressed at E14.5, increased at E16.5 and its levels were stabilized at the E18.5 stage.

PDE4 is a large family of enzymes (4A, 4B, 4C and 4D) encoded by four genes of which *4a*, *4b* and *4d* are expressed in the developing mouse heart and in cardiomyocytes [8,19]. Two mRNA of PDE4 isoforms expressed in the heart were analyzed, *Pde4a* and *Pde4d.* Both transcripts with little differences were expressed at comparable levels during all stages.

PDE5A, a cGMP-specific activity, has a major role as regulator of vascular smooth muscle contraction [15,20]. *Pde5a* mRNA maintained a profile of expression similar along all stages.

PDE8 is a cAMP specific activity comprising the 8A and 8B isoforms [16]. *Pde8a* mRNA was present at low levels between E14.5 and E16.5 and displayed, similar to *Pde2a*, a great increase from E16.5 to E18.5.

PDE9 isoforms specifically hydrolyze cGMP with the highest affinity among all PDE families, and they are expressed in cognitive-relevant regions of the brain [17,21]. *Pde9* mRNA showed a constant level at all stages of heart formation.

Taken together, the mRNA expression of PDE isoforms can be grouped into mRNAs that are present at approximately the same level at any stage of heart development (*Pde1a*, *Pde3a*, *Pde4a* and *Pde5a*), mRNAs that tend to decrease with time (*Pde4d* and *Pde9*) and transcripts that are clearly upregulated with time (*Pde1c*, *Pde2a* and *Pde8a*).

### 2.2. Variation of PDE Expression Occurs at Protein Levels in Embryonic and Fetal Heart

Since no data are available regarding the translation of mRNA of PDE isoforms in the prenatal heart, Western blot (WB) analyses were performed to assess the presence of PDE isoform immunoreactivity on the same days in which mRNA expression was evaluated.

**Figure 2:**
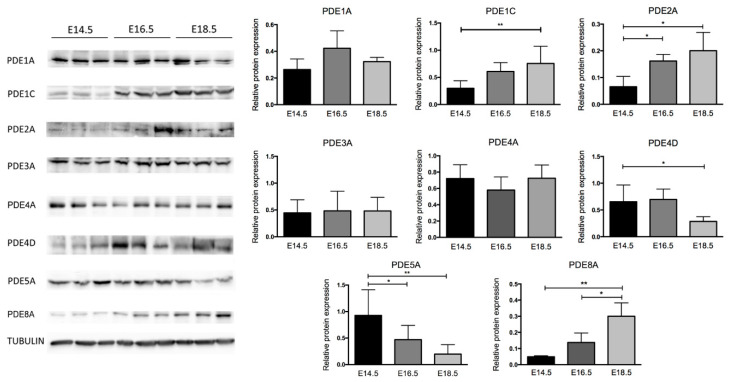
PDE protein expression during heart formation. Representative image of Western blot analysis and relative densitometric analysis of PDE1A, PDE1C, PDE2A, PDE3A, PDE4A, PDE4D, PDE5A and PDE8A proteins normalized with respect to Tubulin protein levels in E14.5, E16.5 and E18.5 mouse hearts. Data represent the mean ± SD of at least three hearts for each embryonic stage. One-way ANOVA was used for statistical analysis, * *p* < 0.05, ** *p* < 0.01.

The WB analysis reported in Figure 2 showed a protein pattern profile that, with the exception of PDE4D and PDE5A, resembled the gene expression profile of Figure 1.

Briefly, PDE1A showed protein levels very similar at all stages of heart formation. Although PDE2A was almost undetectable at E14.5, PDE1C and PDE2A displayed an almost identical progressively increasing profile with the highest protein amounts at E18.5. PDE3A and PDE4A displayed protein amounts similar at all stages evaluated. The level of PDE4D was not modified between E14.5 and E16.5 but showed a significantly detected reduction at E18.5. The level of PDE5A protein greatly decreased from E14.5 to E16.5, reaching its lowest level at E18.5. PDE8A protein amount mirrors the same pattern of PDE2A with a progressive and significant increase during the stages of heart formation. The PDE9 protein was below the limit of quantification of employed methods (data not shown).

### 2.3. Few PDEs Showed Enzymatic Activity Modulation at Embryonic and Fetal Life

**Figure 3:**
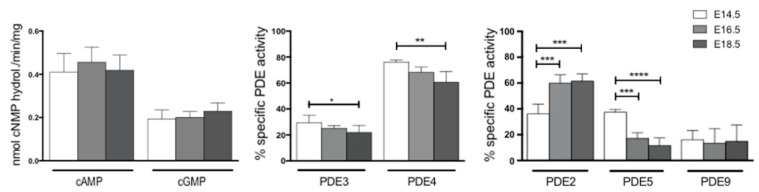
Total and specific PDE enzymatic activity during heart formation. Total cAMP and cGMP hydrolyzing activity from heart homogenates at E14.5 (white columns), E16.5 (light grey columns) and E18.5 (dark grey columns). PDE3- and PDE4-specific activities are reported as a percentage of total cAMP hydrolyzing activity evaluated after adding a fixed dose of the specific inhibitor milrinone and rolipram, respectively (see Materials and Methods). PDE2, PDE5 and PDE9 specific activities as a percentage of the total cGMP hydrolyzing activity evaluated after adding a fixed dose of the specific inhibitor BAY 60-7550, sildenafil and PF04449613, respectively (see Materials and Methods). Data represent the mean of three independent experiments ± SD. Each experiment was performed with a pool of 5 hearts per stage. One-way ANOVA was used for statistical analysis. * *p* < 0.05, ** *p* < 0.01, *** *p* < 0.001, **** *p* < 0.0001.

In the prenatal heart, the levels of cAMP and cGMP hydrolytic activities are stable during development (Figure 3).

Since specific inhibitors for many PDE isoforms are available, it was possible to measure the enzymatic activity of PDE2, 3, 4, 5 and 9 at different stages of heart development. cAMP-PDE activity assays were performed in cardiac homogenates in the presence of 10 μM milrinone, the specific inhibitor of PDE3, and 30 μM rolipram, the specific inhibitor of PDE4. The results obtained showed that almost 75% of the total cAMP hydrolytic activity in the prenatal heart is due to PDE4 and the remaining 25% to PDE3 activity (Figure 3).

PDE3 activity decreased with time, reaching statistical significance at E18.5, while PDE4 decreased more extensively compared to PDE3, showing a 15% decrease from E14.5 to E16.5, whereas the reduction was 20% from E14.5 to E18.5.

cGMP-PDE activity assays were also performed in cardiac homogenates in the presence of 0.1 μM BAY 60-7550, the specific inhibitor of PDE2; 0.1 μM sildenafil, the specific inhibitor of PDE5; or 1 μM PF04449613, the specific inhibitor of PDE9.

In the prenatal heart at E14.5, almost 80% of the total cGMP hydrolytic activity was divided at the same extent between PDE2 and PDE5, and the remaining 10% was due to PDE9 (Figure 3). PDE2-dependent cGMP hydrolytic activity increased from 40% of the total activity at E14.5 to 60% at E16.5 and E18.5. In parallel, there was a significant decrease of PDE5-specific activity from the total 40% at E14.5 to less than 17% at E18.5. PDE9 activity was about 15% of total cGMP activity at every evaluated stage.

**Figure 4:**
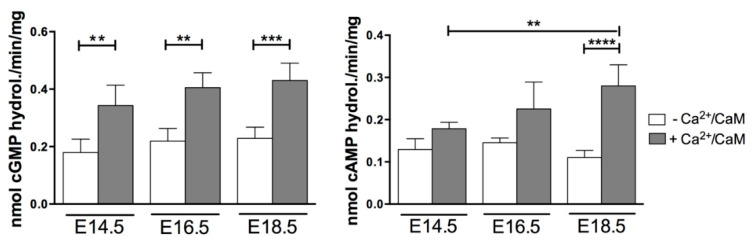
PDE1 enzymatic activity during the heart formation. Total PDE1 activity evaluated with cGMP as substrate in the presence of calcium and calmodulin. PDE1C activity assessed with 0.1 μM cAMP as substrate in the presence of calcium and calmodulin. Data represent the mean of three independent experiments ± SD. Each experiment was performed with a pool of 5 hearts per stage. Two-way ANOVA were used for statistical analysis. ** *p* < 0.01, *** *p* < 0.001, **** *p* < 0.0001.

The PDE1 family comprises 3 genes for PDE1A, PDE1B and PDE1C activities, all able to hydrolyze both cAMP and cGMP [18]. Its hydrolytic activity was evaluated due to its unique property of being stimulated by Ca^2+^/CaM binding. The three isoforms displayed the same affinity for cGMP hydrolysis [22]; therefore, the total amount of PDE1-stimulated activity was first assessed giving cGMP as substrate. As shown in Figure 4, cGMP hydrolyzing activity was stimulated two-fold in the presence of calcium and CaM, showing a constant level independent of the developmental stage.

Since there are no specific inhibitors to PDE1 isoforms but PDE1C has a higher affinity for cAMP with respect to the other two isoforms [23], its hydrolyzing activity was evaluated in the presence of 0.1 μM cAMP (Figure 4). The results obtained showed a similar Ca^2+^/CaM stimulation in both E14.5 and E16.5 hearts and a significant stimulated increase at the E18.5 prenatal heart stage.

## 3. Discussion

The present study characterized the developmental pattern expression and enzymatic activity of PDEs at different stages of embryonic and fetal murine heart.

PDEs are evolutionary-conserved enzymes that, through the hydrolysis of cAMP and cGMP nucleotides, regulate the intracellular transduction signal of many hormones and growth factors involved in cardiac biology [7]. However, few data are available on the levels of cyclic nucleotides in the developing heart. Thakkar and Sperelakis assessed the cAMP and cGMP basal level content during chicken heart development [24]. Their data showed that the cAMP content was highest at early stages of heart development, decreasing with time, while the basal cGMP content was lowest early and increased at a later stage. It has been suggested that the adverse cAMP and cGMP basal content profile might be either due to a decrease of adenylyl and guanylyl cyclase activity or to an increase in PDE activity [24,25]. On the contrary, in isolated mouse cardiomyocytes with increasing age of development, it has been reported that cAMP content increased [26]. Here we report data showing that basal levels of cAMP and cGMP hydrolyzing activity did not change in the developing heart, ruling out the possibility that cyclic nucleotide basal level differences could be due to a modification of the heart cells ability to degrade them. In addition, these data indicated the existence of an equilibrium between the two cyclic nucleotides at least during heart developmental stages.

Previous data obtained by RT-PCR demonstrated that nine PDEs are expressed in the mouse developing heart [8]. These results were not only confirmed and extended by qRT-PCR, WB analyses and activity assays from this study, but we also showed that among the evaluated PDEs families, *Pde1C*, *Pde2A*, *Pde4D*, *Pde5A* and *Pde8A* displayed significant and never-described-before changes in expression in embryonic and fetal murine heart development. In particular, PDE1C, PDE2A and PDE8A activities showed a progressive significant increase while PDE4D and PDE5A showed a progressive decrease during heart formation. Moreover, we found that PDE2A and PDE5A accounts, with an increasing-decreasing adverse profile, respectively, for nearly 80% of the total cGMP hydrolyzing activities, while PDE3 and PDE4 accounts for more than 90% of the total cAMP hydrolyzing activity (Figure 3).

These data suggested the involvement of these activities during the construction of the main frame of the heart, while the other PDEs must have a role in specific modulated-adjusting functions to complete the transition from the embryonic to the final fetal organ.

In detail, we will describe the single-analyzed PDEs to elucidate, according to published data and to the results shown in this paper, the role played by each of them during the process of heart development.

PDE1 is a Ca^2+^/CaM-stimulated phosphodiesterase family comprised of three genes for PDE1A, PDE1B and PDE1C activities, all able to hydrolyze both cAMP and cGMP [18].

The activity assay with cGMP as a substrate and Ca^2+^/CaM, detecting together the three PDE1 stimulated activities, revealed a constant basal level throughout heart development. On the contrary, the Ca^2+^/CaM-stimulated activity specifically associated with PDE1C at low concentration of cAMP [22] showed the same increase observed at mRNA and protein levels with the highest amount at E18.5, indicating that PDE1C might play a major role in the fetal heart rather than in the embryonic heart.

In fact, although PDE1C mutant mice do not present any cardiac alterations in physiological conditions, the genetic ablation of this isoform, expressed exclusively in cardiac myocytes, was associated with cardio-protection [11].

In line with the cell cycle exit of cardiomyocytes shortly after birth to terminal differentiation [27], the increased expression of PDE1C observed during heart development could be associated with PDE1C involvement in the control of cell proliferation as demonstrated in different studies. In particular, PDE1C plays a critical role in regulating the stability of growth factor receptors known to mediate important signaling pathways during heart formation [28].

The PDE2A gene gives rise to three isoforms localized in different cellular compartments, and they are all able to hydrolyze cAMP and cGMP at a similar rate [29]. PDE2A expression markedly increased at mRNA and protein levels at E18.5, accounting for 60% of the total cGMP hydrolyzing activity of fetal heart.

The importance of PDE2A in heart development is evident from its knockout at the embryo stage, which leads to severe cardiac alterations such as incomplete intraventricular septum, ventricular enlargement and hypertrabeculation before death takes place at E15.5 [9]. Moreover, PDE2A is the only PDE shown to be localized in mitochondria, where it is assembled in a multiprotein signaling complex with a specific mitochondrial adenylyl cyclase regulating the activity of the respiratory chain [30]. We cannot exclude that the increase in PDE2A during development could also be associated with the mitochondrial biogenic surge that occurs in cardiomyocytes at birth [31].

PDE5A, which specifically degrades cGMP, showed the highest protein level and hydrolyzing activity at E14.5 and the lowest levels at E18.5 stage.

PDE5A displays reduced levels in the adult heart under physiological conditions and increased levels in several pathological conditions; these differences with its levels during cardiac development suggested a correlation between its expression and hypoxic condition. In fact, at birth, the embryo undergoes a change from hypoxia to normoxia [31], while in the adult heart tissue, hypoxia seems to be one of the common features in cardiovascular disorders [32].

PDE4 is a large family of enzymes (PDE4A, 4B, 4C and 4D) of which A, B and D are expressed in the developing mouse heart, in cardiomyocytes and in murine cardiac tissue at comparable levels [8,13,19]. Despite the high amounts of PDE4 activity, the pharmacological inhibition and the genetic ablation of the PDE4A isoform in mice did not induce any cardiac alterations in animals at six months of age [33]. Conversely, the different phenotypes induced by the genetic ablation of PDE4D is probably due to their different subcellular localization and macromolecular complex interaction. Indeed, PDE4D knockout mice presents a progressive cardiomyopathy with a loss of β-adrenergic signaling in cardiac myocytes and heart failure after myocardial infarction [14,34].

In the developing mouse heart, the *Pde4d* mRNA expression is not modified over time, whereas, at the protein level, PDE4D showed a significant decreased expression at E18.5 (Figure 2). The decrease at the protein level is in agreement with the cAMP-hydrolytic activity decrease observed at the E18.5 stage while no changes were detected in the 4A isoform at the stages examined.

It is well known that the cardiac autonomic nervous system modulates physiological cardiac functions such as heart rate and contraction force. The first signs of cardiac innervation during development are found in mouse dorsal mesocardium at E10.5 [35]. It has been reported that the heart rate of mice increases postnatally [36,37], mainly through the action of sympathetic system. One can speculate that the decreased expression of PDE4D in E18.5 heart could sustain sympathetic activity which in turn induce an increase in cAMP signaling through β-adrenergic receptor activation. In fact, detailed studies demonstrated the greatest role of PDE4D in the control of cAMP generated by β-adrenergic stimulation [6,38].

Another cAMP-specific PDE showing a significant alteration of expression during cardiac development is PDE8A; both mRNA and protein levels significantly increased from E14.5 to the E18.5 stage of heart formation. Although the PDE4 family has a dominant role on the control of cAMP produced following β-adrenergic receptor stimulation, PDE8A is also involved in this pathway. Studies in isolated ventricular myocytes from *Pde8a* knockout hearts have demonstrated that this enzyme regulates Ca^2+^ signaling during β-adrenergic stimulation, suggesting that it could protect against cardiac arrhythmias [16]. However, further studies are required to fully understand the role of PDE8A in heart.

In conclusion, this study showed that there is a modulation of PDEs’ expression at different stages of heart formation which tends to keep constant the total hydrolyzing activity of the heart. The expression of multiple PDE isoenzymes degrading cAMP/cGMP over a wide range of concentrations, and whose activity is dynamic due to PDE regulation by numerous intracellular signals, contributes to the very complex scenario in the regulation of cyclic nucleotide pathways during cardiac development. An additional and important property of the PDE system is its ability to operate in discrete subcellular compartments through macromolecular complexes organized by scaffold proteins. This organization creates high concentrations of specific PDEs which regulate distinct pools of cyclic nucleotides and, in turn, specific cellular processes [39].

Further studies will be necessary to better understand the localization and subcellular functions of different PDEs at different stages of heart formation and to verify if dysregulation of these patterns of expression could be involved in cardiac malformations during the intrauterine life.

## 4. Materials and Methods

### 4.1. Mouse Husbandry and Embryos Collection

All the animal procedures conformed to the Directive 2010/63/EU of the European Parliament on the protection of animals used for scientific purposes, and they were conducted with the approval of the Sapienza University’s Animal Use for Research Ethic Committee and by the Italian Ministry of Health with protocol number DGSAF 24675-A (2013) and 919/2020-PR. Male and female C57BL/6 wild type from eight to twelve weeks of age were mated to generate embryos and fetus mice. The day of vaginal plug was considered as embryonic day 0.5 (E0.5). The animals had access to food and drinking water ad libitum and maintained in a light-dark cyclic of 12-12 h and temperature at 20–25 °C. At 14.5, 16.5 and 18.5 days of heart formation, embryos and fetuses were dissected from the yolk sac after cervical dislocation of pregnant mouse. The embryonic and fetal hearts were collected in phosphate buffer saline and stored at −80 °C for future analysis.

### 4.2. qRT-PCR

Total RNA was isolated from five embryonic heart tissues using total RNA isolation Microprep Kit-BioRad following the manufacturer’s instructions. The RNA was treated with DNase I Zymol and, after, was reverse-transcribed to synthesize cDNA through Maxima H minus reverse transcriptase (Thermo Fischer Scientific, Waltham, MA, USA). For qRT-PCR, samples were amplified in the reaction mixture with PowerUp SYBR green Master Mix (Thermo Fisher Scientific) and specific primers for phosphodiesterases in Thermo Fisher Scientific 7500 Real-Time PCR instrument. For quantification analysis, the comparative threshold cycle (Ct) method was used. The Ct values of each gene were normalized to the Ct value of *Gapdh* in the same RNA sample. The gene expression levels were evaluated by the fold change using the equation 2^−ddCt^. The primers used in the present study are listed in the Table 2.

### 4.3. Western Blot

To evaluate the protein expression of PDEs during cardiac development, a Western blotting analysis was performed. Protein samples were extracted from embryonic heart and lysed in RIPA lysis buffer 1× (Merck Millipore Darmstadt, Germany) supplemented with protease and phosphatase inhibitors. The protein concentration was measured by Bradford method. An amount of 30 μg of protein was electrophoresed in 8–10% SDS-page gels and transferred onto nitrocellulose membrane. The membrane was immersed in 5% *v*/*v* blocking milk (TBST + 5% non-fat milk) for 1 h at room temperature and incubated in primary antibodies (1:500 *v*/*v* in TBST + 5% *w*/*v* BSA (bovine serum albumin) buffer) for phosphodiesterase detection (PDE1A FabGennix #PDE1A-101AP; PDE1C FabGennix #PD1C301AP; PDE2A Abcam #ab140650; PDE3A Santa Cruz #sc-20792; PDE4A Proteintech #16226-1-AP; PDE4D Invitrogen #PA5-79795; PDE5A Santa Cruz #32884; PDE8A Sigma Aldrich #HPA007722) overnight at 4 °C. After, the membranes were washed in TBST and incubated in appropriated horseradish peroxidase (HRP)-conjugated secondary antibodies (1:10,000 *v*/*v* in TBST) for 1 h at room temperature. Chemiluminescent images of immunodetected bands were recorded with the Syngene G-box system (Syngene Bioimaging, India) and immunoblot intensities were quantitatively analyzed using ImageJ Software (NIH, Bethesda, MD, USA).

### 4.4. PDEs Activity Assay

Embryonic hearts were homogenized using a glass homogenizer (15 strokes, 4 °C) in 20 mM Tris-HCl buffer pH 7.2 containing 0.2 mM EGTA, 5 mM β-mercaptoethanol, 2% *v*/*v* antiprotease cocktail (Sigma–Aldrich, St. Louis, MO, USA), 1mM PMSF, 5mM MgCl2, 0.2% *v*/*v* Triton X-100. The homogenates were centrifuged at 14,000× *g* for 30 min at 4 °C.

PDE activity was measured on the supernatant with the method described by Thompson and Appleman [40] in 60 mM Hepes pH 7.2, 0.1 mM EGTA, 5 mM MgCl2, 0.5 mg/mL bovine serum albumin (BSA) and 30 mg/mL soybean trypsin inhibitor in a final volume of 0.15 mL. Ca^2+^-calmodulin stimulation was determined in the presence of 1 mM CaCl_2_ and 3 μg/mL calmodulin. The reaction was started by adding tritiated substrates at a final concentration of 1 μM [^3^H] cAMP or [^3^H] cGMP, and 0.1 μM [^3^H] cAMP for PDE1C detection. The reaction was stopped by adding 50 μL of 0.1 N HCl and then neutralized with 50 μL of 0.1 N NaOH in 0.1 M Tris–HCl pH 8.0. Subsequently, 25 μL of 2 mg/mL of 5′-nucleotidase (snake venom from Crotalus atrox; Sigma-Aldrich) in 0.1M Tris–HCl pH 8.0 were added. Samples were gently mixed and incubated at 30 °C for 30 min to allow complete conversion of 5′-nucleotide to its corresponding nucleoside. Unhydrolyzed cyclic nucleotide and the corresponding nucleoside were separated by DEAE-Sephadex A-25 columns. The eluate was mixed with ULTIMA GOLD scintillation liquid (PerkinElmer) and counted on a Tri-Carb 2100TR Liquid Scintillation Counter (2000CA; Packard Instruments, Meriden, CT, USA).

To evaluate the enzymatic specific activity of each PDEs, the specific inhibitors were added to the reaction mix at the following concentration: 10 μM milrinone (PDE3 inhibitor), 0.1 μM BAY 60-7550 (PDE2 inhibitor), 30 μM rolipram (PDE4 inhibitor), 0.1 μM sildenafil (PDE5 inhibitor) and 1 μM PF04449613 (PDE9 inhibitor). The percentage of each PDE was calculated comparing the difference between the total PDE activity and the residual PDE activity assayed in the presence of the specific inhibitor with respect to the total PDE activity fixed as 100%.

### 4.5. Statistics Analysis

The data obtained were statistically analyzed using the GraphPad Prism 5.0 (GraphPad software, San Diego, CA, USA). Comparison among groups was performed with the one-way ANOVA or two-way ANOVA and multiple comparisons by Tukey test. A *p* value of <0.05 was considered statistically significant; all data were presented as mean ± SD.

## Figures and Tables

**Table 1:** Overview of PDE isoforms involved in cardiac functions.

Phosphodiesterase (PDE) Family	Gene	Substrate/ Regulation	Transgenic Model
PDE1	1A, 1B, 1C	cAMP/cGMP Ca^2+/^CaM stimulated	*Pde1a* knockout: lowest blood pressure, increased heart rate, elevated ejection fraction [10]. *Pde1c* knockout: protected from TAC-induced cardiac dysfunction; antagonize cardiac myocyte hypertrophy and death [11].
PDE2	2A	cAMP/cGMP cGMP-stimulated	*Pde2a* knockout: embryonal death, congenital heart defect [9].
PDE3	3A, 3B	cAMP/cGMP cGMP-inhibited	*Pde3a* overexpression: decreased heart rate and cardiac contractile function; antiapoptotic effect in adult cardiomyocytes [12].
PDE4	4A, 4B, 4C, 4D	cAMP	*Pde4a* knockout: no cardiac alteration [13]; *Pde4d* knockout: age-related cardiomyopathy; arrhythmia [14].
PDE5	5A	cGMP	*Pde5a* overexpression: adverse cardiac remodeling after myocardial infarction; enhanced hypertrophy, reduced contractile function [15].
PDE8	8A, 8B	cAMP	*Pde8a* knockout: increased Ca^2+^ transient and current during β AR stimulation [16].
PDE9	9A	cGMP high affinity	*Pde9a* knockout: cardio-protected after sustained pressure overload [17].

**Table 2:** qRT-PCR primers.

Gene	Forward Primer	Reverse Primer
*Pde1a*	5′AGGTATCATGCACTGGCTCA 3′	5′GAGCGGTCGTTGTACAGAAT 3′
*Pde1c*	5′ATGGGGATGATGCTTAGGAG 3′	5′ CAATGCTTCGATTACAGCCG 3′
*Pde2a*	5′ ACCGAAAGATCCTGCAACTG 3′	5′ TTCTCCCAGCACTTTGTCTC 3′
*Pde3a*	5′ AGAATCCATGCCACCGATGT 3′	5′ CCCATGTGTCCGTGTGTAAA 3′
*Pde4a*	5′ TGCTGCAAGAGAACTGC 3′	5′ AGGGTCATGTGCTTGGACAT 3′
*Pde4d*	5′GCCTCTGACTGTTATCATGCAC3′	5′ GCAGCATGGATGTTGTTGTG 3′
*Pde5a*	5′ ATCCATGGACTCATCTCTGC 3′	5′ GCTTCCTCCAATGTTGAACC 3′
*Pde8a*	5′ TCAGAGTGTGCAATGGCAAC 3′	5′GTCCATCGAATGTTTCCTCC 3′
*Pde9*	5′ CTACGAGGAGCTGAAGCAGC 3′	5′ AGTTTGGAGGAGAATGGCCT 3′
*Gapdh*	5′ GTGAAGGTCGGTGTGAACG 3′	5′ ATTTGATGTTAGTGGGGTCTCG3′

## Data Availability

Not applicable.

## Abbreviations

E.	Embryonic day
cAMP	Cyclic adenosine monophosphate
cGMP	Cyclic guanosine monophosphate
AC	Adenylyl cyclase
NO	Nitric oxide
PDE	Phosphodiesterase
Ca^2+^	Calcium
CaM	Calmodulin
TAC	Transverse aortic constriction
